# Covaxin: An overview of its immunogenicity and safety trials in India

**DOI:** 10.6026/97320630017840

**Published:** 2021-10-31

**Authors:** Rohit Sharma, Swati Tiwari, Aparna Dixit

**Affiliations:** 1 School of Biotechnology, Jawaharlal Nehru University, New Delhi - 110067, India

**Keywords:** Covaxin, BBV152, COVID-19, animal and human trial, SARS-CoV-2 vaccine variants

## Abstract

The spread of severe acute respiratory syndrome coronavirus 2 (SARS-CoV-2) has led to a global coronavirus disease-19 (COVID-19) pandemic. Several vaccine types, such as inactivated, viral vector-, or mRNA-based, have received approval against SARS-CoV-2. The ability to induceT-helper-1 cell (Th1) responses is desirable from an effective vaccine against this virus. Covaxin (BBV152) is a wholevirion inactivated SARS-CoV-2 vaccine adjuvanted with Algel-Imidazoquinoline (IMDG) molecule, a toll-like receptor (TLR) 7/8 agonist. The mRNA-based vaccine use is hindered because of cold storage requirement, whereas covaxin is stored between 2°C and 8°C, making it suitable for countries with limited resources. The Drug Controller General of India (DCGI) has approved the BBV152 vaccine. Therefore, it is of interest to document known data on BBV152 vaccine phase I, phase II and phase III human clinical trials to evaluate the safety, reactogenicity, tolerance, and immunogenicity of the whole-virion inactivated SARS-CoV-2 vaccine (BBV152).

## Background:

SARS-CoV-2 that causes COVID-19 is an enveloped, singlestranded, positive-sense RNA virus that belongs to the β-genus of Sarbecovirus and is a close relative of SARS-CoV [1]. The WHO Coronavirus (COVID-19) Dashboard on 10 September 2021 reports more than 220 million confirmed cases of COVID-19 with more than 4 million deaths worldwide. In India, more than 33 million cases have been reported, and deaths have surpassed 440 thousands [2]. Bharat Biotech Limited developed the first and only indigenousvaccine- "Covaxin" in collaboration with the National Institute of Virology (NIV), an Indian Council of Medical Research (ICMR) institute [3]. This vaccine against COVID-19 was developed by inactivating the whole-virion SARS-CoV-2 strain NIV-2020-770 (which contains the D614G mutation) with β-propiolactone.The vaccine was developed and is manufactured in the Bio-Safety Level 3 (BSL-3) high containment facility at Bharat Biotech Limited. An inactivated vaccine provides a simple and cost-effective alternative, especially given the challenge of vaccinating a large population, with high efficacy and safety, in a country like India.

## Different phases of BBV152 vaccine trials:

### BBV152 (A-C) vaccine animal trials

A whole-virion inactivated SARS-CoV-2 vaccine candidate (BBV152A-C) was developed against the virus pathogenic and predominant strain-NIV-2020-770.BBV152 was formulated in aluminium hydroxide (Algel) or Algel-Imidazoquinoline (Algel-IMDG). IMDG, a synthetic TLR7/8 agonist in Algel-IMDG, helps stimulate Th1-biased immunity and thus helps in enhancing the immunogenicity and protective efficacy of the vaccine candidate [4]. IMDG has also been used in influenza vaccine formulations to stimulate cell-mediated responses [4]. Preclinical studies in three animal models (mice, rats,and rabbits) demonstrated that antisera from all of them inactivated whole-virion. SARS-CoV-2 vaccine candidates-BBV152A [3 µg + (Algel-IMDG), BBV152B (6 µg + Algel-IMDG), and BBV152C (6 µg + Algel)]-showed 100% seroconversion with high titers of antigen binding and neutralizing antibody(NAbs) responses [5]. In another animal model (hamster), BBV152 immunization induced SARS-CoV-2- specific IgGs or Nabsafter the third-week post-immunization [6]. Further, the BBV152B-IMDG Adjuvant formulation showed an antibody response ten times higher when compared to the antigen alone. As expected for a vaccine adjuvanted with IMDG,aratio between IgG2a and IgG1 greater than 1 indicated immunity biased towards Th1-mediated response [5]. During hamsters immunization and post-challenge phase, BBV152 (A, B,and C) induced a predominantly IgG2 response with an increasing trend of IgG2 response [6]. The absence of any lung infection, lower viral loads, and high titers of neutralizing antibodies post-infection demonstrated the protective efficacy of these three vaccine candidates in immunized hamsters. Of these three candidates, the BBV152A formulation was found to be the mostprotectiveagainst CoV-2 infection [5]. Similarly, vaccinated rhesus macaques had no detectable viral gRNA in lavage fluid, nasal swab, throat swab, and lung tissue seven days postinfection or in broncho alveolar lavage fluid on 5th days postinfection [7]. In addition, no evidence of pneumonia was observed by histopathological examination in vaccinated groups, unlike that observed in the control group [7].

### Phase 1 trial

The studies published by Ella and co-workers presented the results of phase I trial with the vaccine formulation of BBV152 [8]. After animal trials, the three different formulations of BBV152 with Algel-IMDG (2-3 µg), 6 µg with Algel-IMDG, 6 µg with Algel, and the control (Algel-only, no antigen) were examined for safety and immunogenicity in a phase 1 trial. The first dose was administered on day 0, with the booster administered on day 14. A total of 375 individuals were enrolled for thetrial,with 100 individuals assigned to each of the three vaccine groups- one for each formulation, and 75 individuals were randomly assigned to the Algel-only control group. Any vaccine candidate needs to generate high titers of neutralizing antibodies, but it should not induce severe local or systemic adverse reactions. Therefore, enrolled individuals were examined and analyzed for these responses. After the first dose, individuals were analyzed for local adverse reactions such as pain at the injection site and swelling. Solicited systemic adverse reactions such as fever, body ache, fatigue, headache, and nausea or vomiting were examined. Interestingly, a negligible percentage of individuals showed pain at the injection site after the first dose (5 % of the individuals administered with 3 µg and 6 µg Algel-IMDG, 1% of those treated with 6µg Algel, and 3% in the Algel-only control group). Interestingly, only 1% of individuals in the Algel-only control group showed any swelling. Booster administration did not enhance the pain or swelling, and only 1%-2% of participants exhibited mild pain in the groups treated with 3 µg and 6 µg Algel-IMDG and 6 µg with Algel. None of the individuals in the Algel-only control group experienced any pain at the injection site. Mild to moderate systemic adverse reaction was observed in all groups. In the 3 µg Algel-IMDG group, of the 5% of the group, which exhibited a response, 0% suffered from fever orbody ache, 3% suffered from fatigue, and 1% complained ofheadache and nausea or vomiting. A higher percentage (14 %) of individuals exhibited an adverse reaction in the group administered with 6 µg with Algel-IMDG. These reactions included fever (2 %), body ache (2 %), fatigue (3 %), headache (5 %), and nausea or vomiting (2%). In the group immunized with 6 µg with Algel, 8 % of the participants presented with adverse reactionssimilar to that observed in the Algel-IMDG group. This suggests that IMDG's inclusion could partly be responsible for a higher percentage of individuals exhibiting an adverse reaction in the previous group administered with Algel-IMDG. Only 3% of the control group suffered from headaches, and 2% complained of nausea or vomiting. After the second dose, 5%, 1%, 4%, and 0% of participants in the 3µg with Algel-IMDG, 6 µg with Algel-IMDG, 6 µg with Algel, and the Algel-only control group, respectively,displayed a systemic reaction like fever, body ache, or fatigue.

IgG titers were examined after two weeks of the booster dose to see if the immune system of enrolled individuals responded appropriately. Anti-nucleoprotein, anti receptor binding, and anti-spike IgG titers were increased in both the groups administered with 3 µg and 6 µg of Algel-IMDG. In addition, the mean isotyping ratios (IgG1/IgG4) were found to be greater than 1 in all vaccinated groups, which was indicative of a Th1 biased immune response.The extent to which antibodies can neutralize SARS-CoV-2 was determined in vitro by a microneutralization assay after the second dose and expressed as a microneutralization titer (MNT). No significant difference was noted in the MNT50 of the individuals vaccinated with the 3 µg and 6 µg Algel-IMDG groups (87.9 % and 91·9%, respectively). However, slightly lower MNT50 (82.8 %) was observed in the 6 µg Algel group. As expected, the control group had an MNT50 of only 8%. On day 28, 2 weeks after the second vaccination in all groups,the paired serum samples (n=50) and neutralizing antibodies were analyzed for neutralizing antibody responses via a microneutralization assay (MNT50) and a plaque reduction neutralization test (PRNT50), respectively. Interestingly, similar neutralization responses were obtained for MNT50 and PRNT50 assays with homologous and heterologous strains, making these vaccine candidates have broader application.

In the phase 1 trial, between 92%-97 % of individuals were followed for the long term, i.e., more than 3 months after the booster dose (92% administered 6µg with Algel, and 97% and 95 % administered with the 3 µg and 6 µg of with Algel-IMDG. On the other hand, only 69 % of the control group individuals administered with Algel were followed for 104 days postadministration of the 2nd dose.

These studies revealed relatively lower geometric mean titers (GMTs) of MNT50 at 3 months from the second dose than that observed on day 28. The reported MNT50 ranged between ∼40 to ∼70 % in the three experimental groups, i.e., the group administered with 3 µg and 6 µg of the vaccine with Algel-IMDG (39.9 % and 69.5%, respectively) and the group administered with 6 µg of formulation with Algel. The control group showed significantly lower MNT50 at 20.7. In addition, seroconversion based on MNT50 in the three experimental groups ranged between ∼73 % to ∼81% (73·5% and 81.1% in the groups given 3 µg and 6 µg vaccine with Algel-IMDG and ∼73 % in the experimental group given 6 µg with Algel group). These data indicated significantly high GMTs in the group administered with the higher dose of vaccine (6 µg)-IMDG relative to the 3 µg in the two Algel-IMDG groups. There were no significant differences in GMTs between 2 weeks after and three months after the second dose across all the vaccine groups. After four weeks, the phase 1 and 2 GMT (MNT50) ratio was reported to be 1.9 for the second dose of 6 µg with Algel-IMDG participants.

In addition to generating a high titer of protective antibodies, memory cells generation is one of the most important criteria for a promising vaccine. Therefore, T cell memory response from different groups was analyzed using peripheral blood mononuclear cells (PBMCs) collected from a subset of phase 1 participants at day 104. The BBV152 formulations with Algel-IMDG showed an increase in the frequency of effector memory CD4+CD45RO+ T cells and CD4+CD45RO+CD27+ T cells compared to pre-vaccination (day 0) samples, which indicates the development of T-cell memory response. These results clearly indicated that the vaccine formulations were capable of generating a T-cell memory. However, the period of 104 days is too short to assess adequate T-cell memory response, and the trial warranted that these individuals be subjected to the same analysis after a more extended period, as is done for other vaccines.

The cell culture supernatant of the PBMCs collected three months after the second dose, stimulated with the antigen for 6 days, was subjected to secreted IgG antibodies titer analysis. All the three experimental groups (3 µg and 6 µg with Algel-IMDG and 6 µg with Algel showed significantly higher IgG antibodies (12.63,16.60, and 19.73, respectively) as compared to prevaccinationtiters (2.33) [8]. Further, various cytokines levels were also measured by Cytokine Bead Array (CBA). The increased level of Th1-biased cytokines (IFN-γ, TNF-α, and IL-2) supported the generation of a T-cell-dependent memory response. On the other hand, no or negligible Th2 cytokines (IL-4) and IL-17A cytokine levels were observed, whereas IL-6 cytokine levels were observed in the 3 µg and 6 µg of BBV152-Algel-IMDG formulations, predicted to be due to the activation of both T & B cells [9].

### Phase 2 trials:

Subsequent to successful completion of phase 1 trial and effective outcomes with Algel-IMDG formulations, two doses of Algel-IMDG formulations (3 µg and 6 µg) were therefore selected for the phase 2 trial to examine their immunogenicity and safety with the first dose administered on day 0 and the second dose on day 28 [9]. In this trial, 380 individuals were enrolled; 190 individuals were assigned randomly to each of the two vaccine groups [Algel-IMDG adjuvantedBBV152 (3 µg and 6 µg)]. After dose 1 (days 0-7), local adverse reactions such aspain, itching, redness at the injection site, weakness, and stiffness in the injection arm were analyzed. The individuals were also examined for systemic adverse reactions such as fever, body ache, fatigue, headache, malaise, and rashes. After the first dose, only 3-4% of individuals in the two groups reported pain at the injection site, and only 2% in each group presented with itching and redness at the injection site. A similar percentage of individuals experienced mild pain at the injection site after the second dose (4% and 3% in the groups administered with the 3 µg and 6 µg of vaccines with Algel-IMDG, respectively).

The systemic adverse reaction was also seen in all groups at mild to moderate levels. For example, in the 3 µg with Algel-IMDG group, of the 5% that presented reactions, 2% exhibited fever and body ache, 2% reported malaise, and 1% suffered from headaches. In the 6 µg with Algel-IMDG group, 10% of the participants reported a reaction, 5% withfever, 2% with body ache, 1% with malaise, 1% with headache, and 1% with weakness. After the second dose,9 % of the 3 µg with Algel-IMDG group and 6% of the 6 µg with Algel-IMDG group showed systemic reactions like fever, body ache, weakness, and malaise.

GMTs from the Plaque Reduction Neutralization Test (PRNT50) values were0.1 in both groups on day 0, and increased to 100.9 and 197.0 on day 56 in the 3 µg with Algel-IMDG group and 6 µg with Algel-IMDG group, respectively. GMTs of microneutralization assay (MNT50) at day 56 were 92.5 and 160.1 in the 3 µg and 6 µg Algel-IMDG group, respectively. Similar to the phase 1 trial, seroconversion based on PRNT50 on day 56 was reported at an average of 92.9% among 184 participants in the 3 µg with Algel-IMDG group and an average of 98.3% in 177 participants in the 6 µg with Algel-IMDG group. Seroconversion based on MNT50 at day 56 was determined to be 88.0% and 96.6 % in 184 and 177 participants in the groups vaccinated with 3 µg and 6 µg with Algel-IMDG group, respectively. Seroconversion rates and GMTs across three age groups (≥12 to <18 years, ≥18 to <55 years, and ≥55 to ≤65-years) and between both sexes were similar. IgG antibody titers (GMTs) against all antigenic epitopes (spike glycoprotein, receptor-binding domain, and nucleocapsid protein) were detected after the administration of both doses. Anti-spike glycoprotein IgG GMT at day 56 was 10413.9 in the 3 µg with Algel-IMDG group and 9541.6in the 6 µg with Algel-IMDG group. In addition, both the groups showed similar antispike glycoprotein, anti-receptor-binding domain, and antinucleocapsid protein GMTs. On day 42, the anti-spike isotype means ratios (IgG1/IgG4) were 2.4 and 2.2 in the groups vaccinated with 3 µg and 6 µg BBV152 with Algel-IMDG, respectively. In addition, the Th1/Th2 cytokine ratio indicated a bias towards a Th1 cell response at day 42.

### Phase 3 Trials:

Details of Phase 3 Covaxin trials have been given in a study conducted by Ella and co-workers [10]. The double blind and randomised phase 3 trials were conducted to evaluate the efficacy, safety, and immunological lot consistency of 6µg BBV152-Algel-IMDG formulation at multiple test centers in India. The study was done with a vaccinated group (n=12,221) and a placebo group (n = 12,198). In a case-driven analysis, 103 cases showed low to mild symptoms (included pain at the injection site and swelling), 24 occurred in the vaccine group, and 106 in placebo recipients, and the efficacy was 77.8%. Sixteen individuals showed severe Covid-19 symptoms (fever,fatigue/malaise, myalgia, body aches, headache,nausea/vomiting, anorexia, chills, generalized rash, and diarrhea), only one in the vaccinated groupmet the severe symptomatic COVID-19 case definition. Thus, the efficacy was determined to be 93.4%. Thus, the trial demonstrated BBV152's high efficacy against symptomatic and asymptomatic COVID-19 variants. On the other hand, a total of 15 deaths were reported, five in BBV512 recipients and 10 in the placebo group. The five deaths were unrelated to covid vaccination; they were due to cerebellar hemorrhage, hemorrhagic stroke, and ovarian cancer with metastases, sudden cardiac death, and COVID-19. Further, the immune response of the three different lots of BBV512 and the placebo were evaluated using a wild-type virus microneutralization assay (MNT50). The GMTs reported were 130.3, 121.2, 125.4, and 13.7 for BBV512 lot 1, 2, 3, and placebo, respectively, demonstrating a consistent immune response in different lots of the BBV512. In addition, the IgG titers to all three epitopes, S1 protein (9742 EU/mL), RBD (4124 EU/mL), and N protein (4161 EU/mL), were also determined to be consistent for all the three different lots.

## Efficacy of Covaxin against SARS-CoV-2variants

From January to April 2021 in India, various variants of SARSCoV-2 were reported. Some, such as B.1.1.7 (United Kingdom)[11], B.1.351(South Africa) [12], B.1.1.28 (Brazil P1, P2), and B.1.617.2, were considered variants of concern. Therefore, it is important to analyze the efficacy of the BBV152 vaccine against all these variants and is briefly discussed below.

## B.1.1.7 variant

The UK variant B.1.1.7 variant is also known as an alpha variant. The UK-variant is shown to have various mutations on the spike receptor-binding domain (RBD), which aid its attachment to the angiotensin-converting enzyme 2 (ACE2) receptor on the surface of human cells [13]. The PRNT was done with sera collected (n=38) from BBV152 vaccine recipients against hCoV-19/India/2020770 (homologous), hCoV-19/India/20203522 (heterologous UK strain), and hCoV 19/India/2020Q111 (heterologous unclassified cluster). The PRNT50 values for all the groups were determined to be the same with no significant difference among different groups. When the median ratio of 50% neutralization of sera was compared with homologous and heterologous UK strains, the value was found to be 0.8 [14]. The PRNT50 data indicate that sera from the BBV152 vaccine recipients could possibly neutralize the UK-variant strains.

## B.1.617 variant

Various B.1.617 variants were reported in the Indian population, such as B.1.617.2, B1617.1, B.617.3, and B.1.1.7. The B.1.617.2 variant, also known as the delta variant, was found to be more deadly and wide spread because of higher transmissibility and potential immune escape. This variant was isolated from the state of Maharashtra in India. This particular variant has shown several spike mutations (T19R, G142D, E154del, A222V, L452R,T478K, D614G, P681R, and D950N) [15]. The neutralization efficacy of the B.1.617 variant was compared with the prototype strain B1 (D614G) and B.1.1.7 variant using sera of BBV152 vaccinated individuals (n=28). The GMT ratio was found to be 1.95 for D614G vs. B.1.617 and 1.84 for B.1.1.7vs. B.1.617 [15]. In addition, another study assessed the neutralization capacity of COVID-19 recovered (n=20) and vaccinated individual sera (n=17) against the particular B.1.617.2 variant of concern compared to the D614G strain. The GMT values were 68.97 and 21.2 for vaccinated individual sera and recovered individual sera, respectively. On the other hand, the GMT ratio of the D614G strain and B.1.617.2 was 2.7 and 4.6 in vaccinated and recovered individual sera, respectively. These data suggest a significant reduction in the neutralization titer for B.1.617.2 compared to D614G in sera of vaccinees and recovered cases. Thus, the data indicate that the neutralizing capacity against variant B.1.617 is the same for sera of COVID-19 recovered individuals (GMT 86.85) and vaccine recipients (GMT 88.48), indicating the potential protective efficacy of the BBV152 vaccine (Covaxin)against the B.1.617 variant.

## B.1.351 variant

B.1.351 is an African variant, frequently referred to as the beta variant, and is also known for higher transmissibility and potential immune escape. A study assessed the neutralization capacity of COVID-19 recovered (n=20) and vaccinated individual sera (n=17) against the B.1.351 variant of concern compared to the D614G (B.1) strain [16].The results indicate the reduction in neutralization titers compared to D614G(B.1) strain in the African variant shows 3.3 fold and 3.0 fold reduction in sera of COVID-19 recovered and vaccinated individuals. Although there is a reduction in neutralization titer, The GMT values were 61.57 and 29.6 for vaccinated individual sera and recovered individual sera, respectively, against African variant. This result indicates BBV152 protective response against a variant of concern B.1351.

## B.1.1.28 P2 variant

The IgG immune response against the P2 variant (GMT of an IgG titer) in 19 sera specimens obtained from recovered cases of COVID-19 was observed to be 794.8 and 4627 for the S1-RBD and the N protein, respectively, and the GMT IgG titer was found to be 2250 with S1-RBD and 3099 with the N protein for Covaxin recipients [17]. In addition, the GMT of the neutralizing antibodies was found to be 337.5 and 175.7 against D614G strain and B.1.1.28.2 variant, respectively in the sera from Covaxin recipients and was 120.1 109.2 for D614G strain and B.1.1.28.2 variant for sera from naturally infected individuals [17]. Thus, this study shows that the Covaxin vaccination significantly boosted the IgG titer and neutralized efficacy against both variants compared to the immunity provided by natural infection.

## Conclusion:

Regulatory approvals have been granted across the world to several vaccines against SARS CoV2. The speed at which the vaccines have been approved for human use is unprecedented. This has been justified because of the scale and morbidity, and mortality experienced across many countries of the world. Vaccines had to be approved for emergency use even though the number of individuals enrolled in each trial was relatively low in many cases like the Covaxin phase 1 trial (n=375) [8], Phase 2/3 clinical trial of COVISHIELD (n=1077 participants) [18], DNA SARS-CoV-2 vaccine (ZyCoV-D) phase I (n=126) [19] and Sputnik V phase 3 trial (n=19 866) [20]. The speed at which Russia and China granted emergency use approvals was met with scepticism and criticized widely. Emergency limited use approvals were granted to Covaxin even before the Phase III results were made public. While the Phase III data showed that it is safe for human use. However, the number of people that participated in the study was low. The vaccine is being used for general use in India and several countries across the world; there is a need to collect and analyze such data carefully. No large-scale adverse reactions have been reported so far, supporting the notion that it is generally safe. Nevertheless, the vaccine's long-term safety needs to be monitored continuously, and deaths resulting after vaccination must be carefully analyzed rather than clubbed under the 'unrelated' deaths. This is difficult to do under the already stretched medical system and many countries with poor resources, but as a large number of people get vaccinated, rare side-effects may be observed, as is the case for several other vaccines like in case of Covaxin, 12% of subjects experiencing commonly known side effects and less than 0.5% of subjects feeling severe adverse events [12]. Further, the time gap between the 1st and 2nd doses of Covaxin was changed, which could be due to continuing studies being carried out with the vaccine. However, no data is available in the public domain regarding this. It is also possible that this decision was felt necessary given India's large population and the rate at which the doses could be produced. Further, there is an urgent need to study the effect of multiple boosters and how long these booster shots could be given. This is necessary as there are doubts about the long-term protective immunity generated in vaccinated individuals. It would be interesting to study if supplementing BBV152 formulations with natural immunomodulators (natural molecules/fractions from medicinal plants) would induce longlasting immunity. COVID-19 pandemic, which has affected the whole world alike, is a challenge to reckon with. Being an RNA virus, the virus genome is likely to mutate more during transmission and propagation in infected individuals. It is not clear whether this will result in the attenuation of the virus or the emergence of more deadly variants. While Covaxin appears to be efficacious against several variants reported so far, how the vaccine will fare with variants that get farther from the parent Covaxin will need to be monitored continuously.
